# Spectral image scanning microscopy

**DOI:** 10.1364/BOE.10.002513

**Published:** 2019-04-22

**Authors:** Franziska Strasser, Martin Offterdinger, Rafael Piestun, Alexander Jesacher

**Affiliations:** 1 Division of Biomedical Physics, Medical University of Innsbruck, Müllerstraße 44, 6020 Innsbruck, Austria; 2 Division of Neurobiochemistry, Biooptics, Medical University of Innsbruck, Innrain 80–82, 6020 Innsbruck, Austria; 3 Department of Electrical and Computer Engineering, University of Colorado, Boulder, CO 80309, USA

## Abstract

For decades, the confocal microscope has represented one of the dominant imaging systems in biomedical imaging at sub-cellular lengthscales. Recently, however, it has increasingly been replaced by a related, but more powerful successor technique termed image scanning microscopy (ISM). In this article, we present ISM capable of measuring spectroscopic information such as that contained in fluorescence or Raman images. Compared to established confocal spectroscopic imaging systems, our implementation offers similar spectral resolution, but higher spatial resolution and detection efficiency. Color sensitivity is achieved by a grating placed in the detection path in conjunction with a camera collecting both spatial and spectral information. The multidimensional data is processed using multi-view maximum likelihood image reconstruction. Our findings are supported by numerical simulations and experiments on micro beads and double-stained HeLa cells.

## 1. Introduction

Lately, there has been an increasing need to acquire spectral information in conjunction with high resolution images. This capability is particularly important to distinguish multiple fluorescent markers simultaneously, even when they present similar emission spectra, such as EYFP and EGFP [1].

One possible technique is confocal spectral imaging (CSI), which combines confocal microscopy and spectroscopy in order to collect high resolution optical sections with speficic color information. Although suitable combinations of color filters can be used to differentiate between emission spectra, this approach is often linked to high signal loss and lack of flexibility, due to the fact that the filters need to be changed for different combinations of markers.

Commercial CSI microscopes usually employ a spectral sensing unit behind the detection pinhole (or fiber), which consists of an image relay containing a dispersive grating or prism and a multi-channel detector. Common systems employ multi-anode photo multiplier tubes (PMT) featuring multiple (e.g. 32) channels, each covering a spectral band of several nanometers. CSI microscopes are usually designed in a way that the image of the detection pinhole on the multi-anode PMT is smaller than the size of an individual detector, because this decouples spectral resolution from the chosen pinhole size [2]. This, however, prevents its combination with Image Scanning Microscopy (ISM) [3, 4]. ISM is an imaging technique that enables the spatial resolution of a confocal microscope with almost fully closed pinhole while essentially all the light reaching the image plane is collected with a detector array, therefore providing bright high-resolution images. After its first experimental demonstration in 2010 [4], ISM has been realized in various ways [5–8], amongst them also all-optical designs [9–14] and a spectrally sensitive Raman ISM [15], which is functionally related to the work presented here.

In this paper, we introduce a route to combine ISM with spectral sensing. This route consists of a specific experimental configuration which physically enables ISM and a matched data processing algorithm. By means of numerical simulations and experiments we prove that our approach is capable of collecting spectral information similar to CSI whilst providing high spatial resolution comparable to ISM. We treat the imaging problem under the more general framework of *engineered Image Scanning Microscopy (eISM)*, which we recently defined as ISM using specifically designed excitation and/or detection pupils in conjunction with matched data processing [16]. eISM thus represents an example for an *integrated optical design* [17], where PSF engineering and data processing work hand in hand to obtain optimal imaging.

## 2. Engineered image scanning microscopy for the measurement of emission spectra

**Fig. 1:**
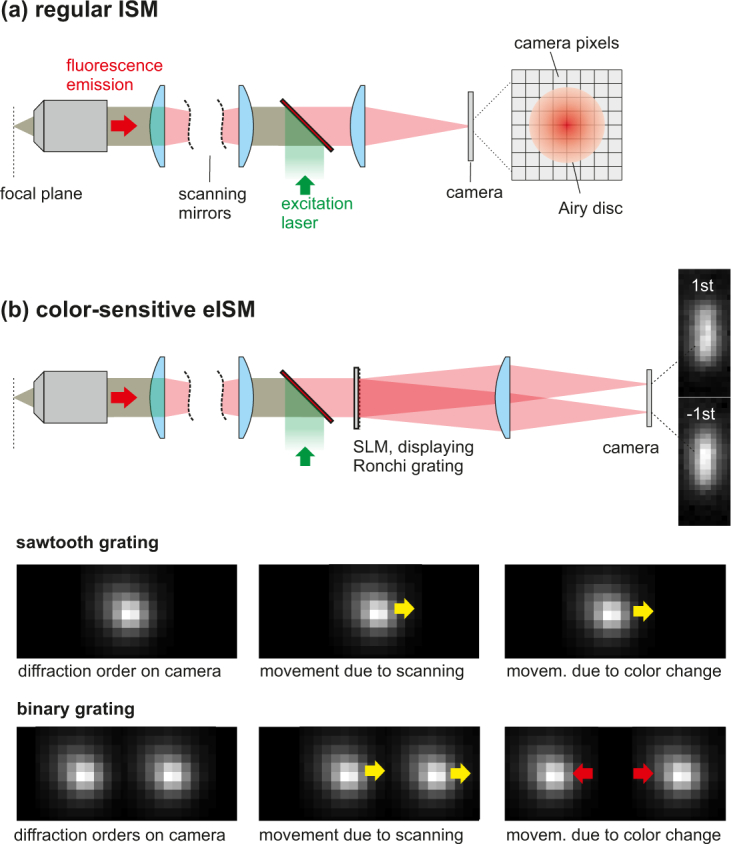
(a) Traditional ISM setup. The signal generated in the excitation focus is imaged onto a pixelated detector. Each pixel *m* records a low-signal but high-resolution confocal image *I_m_*, which are computationally combined to a bright high resolution image of the specimen. (b) Spectrally sensitive eISM setup. A Ronchi phase grating in the detection pupil serves as dispersive element. The 1st and -1st diffraction orders are recorded at each sample scanpoint. The images at the bottom show movements of diffraction orders on the camera for two different scenarios: When the excitation spot is scanned from left to right over a monochromatic point source, all diffraction orders undergo the same movement: They slightly follow the excitation spot along the scanning direction. On the other hand, if the color of the source changes, using a binary grating is advantageous, because the altered distance between the two orders allows for discriminating this case from changes caused by the scanspot-sweep.

Our microscope uses the concept of ISM, which is basically a confocal microscope with camera detection. The ISM principle is outlined in Fig. 1(a)

. Every camera pixel *m* represents an individual confocal detector which records an individual confocal image *I_m_* during a scan. This is why ISM can be considered a multi-view (MV) imaging system. The advantage of ISM compared to a confocal microscope is that these images *I_m_* are sharper, because the pixels are typically small compared to the Airy disc. The optical transfer function is basically that of a confocal microscope with point-like pinhole. At the same time, the light efficiency can be quite high, because no physical pinhole discards light. The idea of ISM, together with an efficient scheme to process the images *I_m_*, was already published in 1988 [3]. More recently, data processing using multi-view deconvolution was proposed [18], which has the advantage that it plays no role whether the point spread functions (PSF) of the individual views are similar (such as in regular ISM) or highly diverse. This fact makes algorithmic multi-view reconstruction a powerful method for the combination of ISM with PSF engineering, which we recently proposed and demonstrated [16, 19].

The use of PSF engineering in microscopy has been considered quite early, with the increase of spatial resolution or depth of field being its main applications [20–24]. In combination with ISM, PSF engineering can be particularly useful, because not only a single but many OTFs can be altered simultaneously and individually, thus opening the door to novel imaging modalities.

A common strategy in PSF engineering is to shape the PSF in a way that it becomes exquisitely susceptible to any property of interest that is sensitive to the interaction with light. In previous publications we focused on measuring a specimen’s 3D structure [16, 19, 25], but also characteristics such as molecule orientation, induced wavefront aberrations or excitation/emission spectra are quantities which could be optimally measured using eISM.

Following this route for the application of measuring emission spectra, we have to engineer a PSF that is sensitive to the emission wavelength. This can be achieved by implementing a grating or prism in the exit pupil of the microscope objective (see Fig. 1(b)). In this aspect, our strategy is analogous to CSI. However, the subtle but important difference is that we have to maintain a fine focus sampling at the detector. Since every pixel takes the role of a pinhole, the system only then physically supports the high spatial resolution of ISM. Unfortunately, this fine sampling imposes a challenge: Because the resolution benefit of ISM relies on measuring subtle focus movements at the camera while the excitation spot sweeps through the sample, it is necessary to disentangle this information from focus shifts induced by wavelength changes. Note that this problem does not exist in common CSI systems where the detector size is much larger than the Airy disc. In other words, we have the problem of sensing 3D information (x,y,*λ*) with a 2D sensor (x,y), a well-known problem in hyperspectral imaging [26] and depth measurements [27].

An important consequence of this fact in view of PSF design is that in addition to providing spectral sensitivity we also have to ensure that the spatio-spectral information is well separable and the image reconstruction problem well-posed. As we outline later, using a prism or blazed grating as dispersive element is not the optimal choice in this regard.

To separate spectral from spatial information we employ a multi-view variant [19] of the Lucy-Richardson algorithm [28, 29]. Basically, the digital post-processing reconstructs a 3D (x,y,*λ*) image from multiple 2D views of the same specimen. Each view *I_m_* is given by a confocal image delivered by an individual detector pixel *m*. Details of the algorithm are reported in Refs [16, 19], which describe its use for reconstructing 3D spatial images from a series of 2D measurements. In fact, the following mathematical considerations are very similar to those in Ref. [16].

The confocal image *I_m_* of detector pixel *m* can be described by a convolution of the spatio-spectral fluorophore distribution ρ(x,y,λ) and its PSF $h_{m}\left(x,y,\lambda \right)$:

$$I_{m}\left(x_{s},y_{s}\right)\propto \left(\rho \;{*}_{3D}\;h_{m}\right)\left(x_{s},y_{s},\lambda_{c}\right).$$ (1)

Here, the coordinates $x_{s},y_{s}$ denote the scanning position and *λ_c_* a specific wavelength. The actual value of *λ_c_* depends on the system settings, but it usually defines the center-wavelength of the detected spectroscopic range. The operator ${*}_{3D}$ denotes a 3D convolution. As each pixel represents an individual small confocal detector, the PSFs *h_m_* can be approximated as

$$h_{m}\left(x,y,\lambda \right)=h_{ex}\left(x,y\right)\cdot \left(P_{m}{⋆}_{2D}h_{\text{det}}\right)\left(x,y,\lambda \right).$$ (2)

*P_m_* is a function describing the pixel shape and can be approximated by a delta-distribution at the position of pixel *m*: $P_{m}\left(x,y\right)\propto \delta \left(x-x_{m},y-y_{m}\right)$. The missing *λ*-coordinate of the excitation PSF *h_ex_* in the equation above means that is has no wavelength dependence. Although in fact it has a single, precisely defined wavelength, this mathematical representation merely accounts for the fact that light of all emission wavelengths share a common excitation wavelength. The operator ${⋆}_{2D}$ denotes a 2D cross-correlation in the spatial plane.

**Fig. 2:**
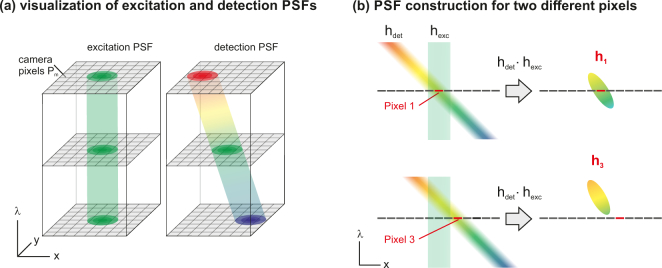
(a) Graphical representation of the excitation and detection PSFs *h_ex_* and *h_det_*. Their spatial shapes are close to Gaussian, their orientations with respect to the wavelength axis straight and sloped, respectively. The steepness of the slope is determined by the period of the grating in the detection path. (b) Visualization of Eq. (2), which describes the formation of pixel-dependent PSFs *h_m_* by spatially translating the detection PSF to the position of pixel *m* and subsequent multiplications with the excitation PSF.

A graphical interpretation of Eq. (2) is given in Fig. 2

. The PSF $h_{m}\left(x,y,\lambda \right)$ is obtained by translating the detection PSF $h_{\text{det}}\left(x,y,\lambda \right)$ to the position of pixel *m* and a subsequent multiplication with the excitation PSF $h_{ex}\left(x,y\right)$, which is assumed to be wavelength-invariant. The figure sketches the process for two different detection pixels in order to show that the resulting PSFs cover different wavelength ranges.

Taking a close look at the shape of the PSFs, we see that they are tilted with respect to the *λ*-axis, which originates from $h_{\text{det}}$ being tilted. Although the multi-view deconvolution takes any PSF shape into account and therefore should ideally be able to remove this x-*λ*-crosstalk, we found that it is still noticeable in the processed data, where it leads to a spatial drift of the final object estimate if one browses through the wavelength channels. This effect is further discussed in the Appendix.

The reason why this crosstalk cannot be entirely removed is that the deconvolution problem is not sufficiently well posed: All PSFs exhibit the same x-*λ*-tilt, which means that there is a lack of diversity from which the algorithm could draw sufficient additional information. In order to improve the situation, we propose to replace the sawtooth grating in the detection pupil by a binary Ronchi phase grating with a phase modulation depth of *π* rad for the center wavelength *λ_c_* and to record the 1st and -1st diffraction orders on the camera, such as shown in Fig. 1(b). The PSFs of pixels covered by the two diffraction orders of the Ronchi grating provide more information: each tilted PSF *h_m_* of a detector pixel in the 1st order has its “counterpart” in a pixel of the conjugated order showing an opposite x-*λ* tilt. The advantage of this strategy is that the space-wavelength-coupling is removed, however at the cost of recording twice as many pixels and a lower light efficiency (both first orders contain 81% of the diffracted light).

A more descriptive way of understanding why the binary grating approach performs better is illustrated in the bottom image rows of Fig. 1(b). If a sawtooth grating is used (first image row), the fine movements of the point response when the excitation spot scans over a fluorophore can be easily confused with a wavelength change of the emitter: In both cases, the point-images move to the right in the shown example. Conversely, if a binary grating is used (second image row), the two scenarios have different effects on the point response: The scanspot-sweep causes a common movement of the diffraction orders, while a color change alters their distance (here the case of a wavelength increase is shown). Consequently, keeping the two cases apart is easier and the deconvolution problem better posed. Notably, an equivalent strategy has been applied for combined position/wavelength measurements in widefield localization microscopy [30].

## 3. Numerical simulations

The performance of our microscope is investigated by numerical simulations using MATLAB. Our model system has the following properties: NA=1.4, refractive indices of immersion oil, coverslip and sample = 1.52, effective pixel size = 60 nm, excitation vacuum wavelength *λ*_0_ = 455 nm. The wavelength-dependent shift of the point-response at the camera is assumed to be 0.27 pixel per nm, which matches the value in our experimental setup. Thus a camera pixel covers a wavelength range of almost 4 nm, which is a similar value to that of commercial CSI systems [2].

PSF simulations were conducted by taking into account vectorial effects (see e.g. [31]), under the assumption of circular excitation polarization and unpolarized fluorescence emission. We further assumed that all polarization directions are detected with equal efficiency.

**Fig. 3:**
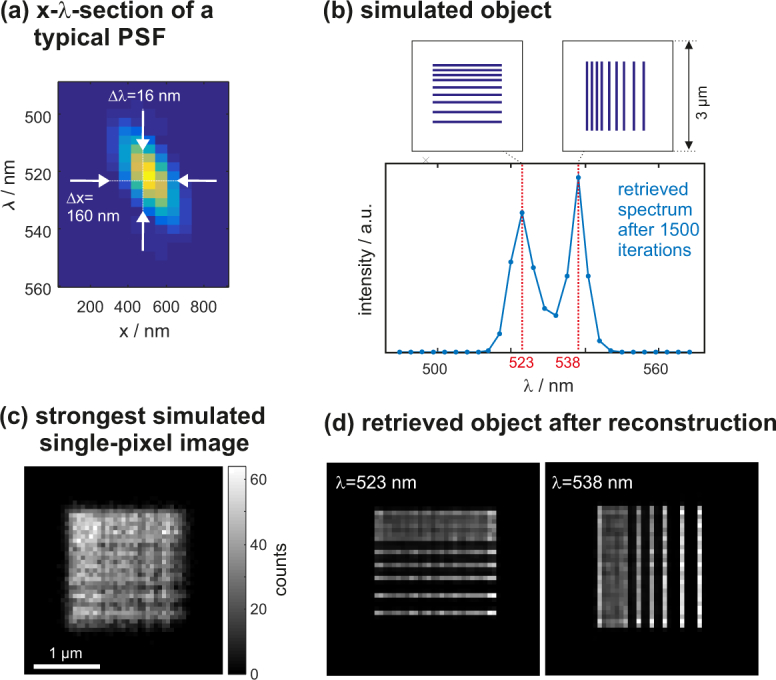
Numerical simulations to assess the imaging properties: (a) x-*λ*-cross section through the simulated PSF of a typical detector pixel. Its FWHM values along the spatial and spectral directions serve as estimate for the achievable resolutions. (b) Properties of object used for simulations. (c) Brightest single-pixel-image with shot noise. (d) Retrieved object after 1500 iterations.

Describing the image formation using 3D spatio-spectral PSFs as presented in the previous section already provides means to estimate the physically supported spatial and spectral resolutions of the imaging system, by taking the full-width at half-maximum (FWHM) of cross sections along the respective directions through the PSFs. A x-*λ*-cross section through the simulated PSF of a particular detector pixel is shown in Fig. 3(a)

. Note that each pixel has a partner-pixel with a PSF that is mirrored about the *λ*-axis but otherwise identical. This ensures an overall symmetry and prevents the positions of features in an image from depending on the emission wavelength. According to the Rayleigh criterion, the transverse spatial resolution is about 160 nm ($\approx \lambda_{0}/3$) along the x-y directions, and the spectral resolution about 16 nm.

To verify if these values are obtainable in practice, we simulate the imaging of a test object (shown in Fig. 3(b)). The assumed object consist of spatially superposed horizontal and vertical line patterns, covering a region of about 1.7×1.7 μm^2^ and emitting at 523 nm and 538 nm, respectively. Their spectral separation thus roughly matches the estimated resolution limit. The smallest spatial line period is 120 nm, which is even below the spatial Rayleigh resolution limit of 160 nm. The size of the canvas containing the object in x-y-*λ*-space is 50×50×27 pixels^3^, equaling 3000×3000×81 nm^3^ at grid increments of 60×60×3 nm^3^.

We consider shot noise and choose the signal strength such that the brightest confocal image (shown in c) collected by any of the detector pixels has an expectancy value of 50 counts per pixel, thus featuring a signal to noise ratio of about 7. This is in accordance with our typical experiments (see Fig. 8(b)). The number of read-out detector pixels (and thus views) is 344, i.e. 172 per diffraction order. However, as explained in the Appendix (data processing section), this large number of views can be quickly reduced to merely 32 using pixel-reassignment [3], which finally serve as input data for the deconvolution algorithm, together with the corresponding PSFs.

Figure 3(d) shows the retrieved object intensity at wavelengths of 523 nm and 538 nm after running the algorithm for 1500 iterations, which takes 7 minutes on a laptop CPU (Intel Core i7-3520M @2.90GHz). The line patterns appear separated along the wavelength direction. The spectral energy distribution is shown in (b). The closest line pairs (120 nm period) cannot be spatially resolved, which is expected due to their distance lying below the resolution limit. The line pairs showing an interspacing of 180 nm, however, appear clearly resolved.

**Fig. 4:**
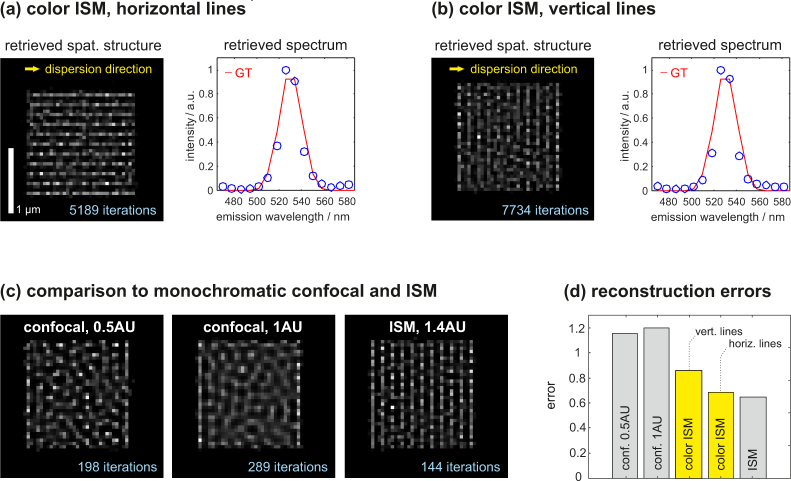
Imaging simulations of grating structures with broader emission spectra at the spatial resolution threshold. (a) and (b) show reconstructions of horizontal and vertical gratings. (c) shows results from monochromatic confocal imaging for two different pinhole sizes as well as ISM. The error bars in (d) express remaining differences between ground truth and reconstructions. Due to remaining spatio-spectral crosstalk, the vertical grating image shows larger errors than the horizontal one, but is still better than the confocal images.

The deconvolution problem is more challenging if a larger number of spectral points shall be retrieved. Then, the spatio-spectral unmixing procedure converges slowly and a residual broadening of spatial sample structures along the dispersion direction (here the horizontal direction) remains. A situation marking the onset of this effect is investigated in Fig. 4

. Here we assume two objects, one consisting of horizontal, the other of vertical lines showing spatial periods close to the resolution threshold of ISM (150 nm) as well as Gaussian emission spectra (*σ*=10 nm) around a center wavelength of 530 nm. The assumed signal strengths are 100k photons in total for the ISM-based methods and 76k(28k) photons for the 1AU(0.5AU) confocal microscopes. Figure 4(a) and (b) show the color ISM reconstructions of both objects. A slight degradation of the vertical line reconstruction compared to the horizontal one is noticeable. The retrieved spectra, however, are almost identical and closely match the ground truth (red line).

In Fig. 4(c) we further compare these results to simulations of monochromatic confocal imaging and ISM. The confocal pinhole diameters measure 0.5 and 1 Airy units (AU), respectively, where the Airy disc diameter is calculated using the average of excitation and peak emission wavelengths. All simulated raw images have been deconvolved using the same algorithm, which stops when the difference between ground truth and reconstruction arrives at a minimum, i.e., when the optimal reconstruction has been obtained. The respective error metric is defined as $\overset{N}{\underset{n=1}{\sum }}|GT\left(n\right)-R_{i}\left(n\right)|$, where *n* denotes the pixel index, *N* the total number of pixels, *GT* the ground truth and *R_i_* the spatial reconstruction after the *i*th iteration. Ground truths and reconstructions containing spectral information are summed up along the wavelength axis prior to error calculation. The final, minimal error values for each reconstruction are shown in the bar plot. The iteration numbers required to achieve optimal reconstructions are stated in the respective images of Fig. 4.

A possibility to improve the spectral ISM reconstruction is to include prior knowledge about the emission color. For instance, if an encompassing bandpass filter is used in the detection path (such as in the experiments introduced in the following section), spectral regions of darkness are known. This information could be used as a constraint in the deconvolution algorithm. Finally, we would like to note that a performance loss of spectral eISM as presented here compared to regular CSI can be easily prevented by introducing a pinhole in a conjugated image plane. From a hardware perspective, the system then practically is a CSI.

## 4. Experiments

We implemented color-sensitive eISM around a home-built confocal microscope. Details about the setup are described in Ref. [16].

The phase grating is displayed on a liquid crystal spatial light modulator (SLM) from Hamamatsu (X10468-01, 800×600 pixels) in the detection pupil. The spectral resolution can be adapted by choosing an appropriate grating period. The used excitation lasers are fiber-coupled and temperature stabilized diodes emitting at 455 nm (Osram PL 450B) and 640 nm (Toptica iBeam smart), respectively. The employed color filters for the blue excitation are parts of a GFP-filter set from Thorlabs (dichroic: *MD 498*, emission filter: *MF525-39*). For the red excitation, the dichroic *H 643 LPXR superflat* from AHF Analysentechnik and emission filter *ET655LP* from Chroma were used. The camera is a Hamamatsu Orca flash 4.0 v2. Recordings are currently taken at about 400 Hz (2 ms pixel dwell time) to prove the concept, i.e. scanning an ISM image of 100 × 100 pixel takes 25 seconds.

To characterize the experimentally obtainable resolution we prepared a glass slide with a mix of two types of fluorescent microbeads from Thermo-Fisher, which exhibit similar diameters and emission spectra (*PS Speck*, 175 nm diameter, dye: “deep red” and *TetraSpeck*, 200 nm diameter, dye: “dark red”). Drops of bead solution were put on a glass slide, air dried, immersed in mounting medium and finally covered with a microscope coverslip.

**Fig. 5:**
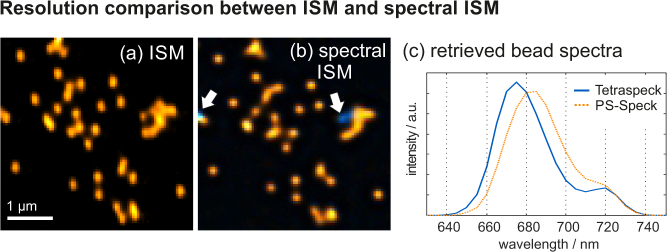
Experimental results from the imaging of fluorescent micro beads using regular (a) and spectral (b) ISM. Along the dispersion direction (horizontal) the resolution of regular ISM is slightly better. The spectral mode, however, was capable to identify individual “TetraSpeck” beads (indicated by white arrows and shown in blue). The retrieved emission spectra are shown in (c).

The first imaging experiment was performed in regular (i.e. monochromatic) ISM mode. For this sake, a blazed grating with a large period of 60 pixels was displayed on the SLM. The grating is sufficient to spatially separate the first from the otherwise disturbing zeroth diffraction order, but does not introduce significant dispersion. The raw data from 45 detectors (covering an approximately circular area of 2 Airy discs) was deconvolved in 50 iterations. The resulting image is shown in Fig. 5(a)

.

Subsequently, the SLM-grating was set to a binary version with periods of 5 and 40 pixels in the horizontal and vertical directions, respectively, such that significant dispersion is introduced. The vertical grating is used to compensate a slight geometric image rotation, thus aligning the diffraction axis with the camera rows. In conjunction with the tube lens (focal length = 200 mm) between SLM and camera, the wavelength dispersion obtained on the sensor was determined to be 6.25 nm/pixel by diffracting a laser beam of known wavelength.

A second scan was taken and the raw data from 234 detectors processed in 100 iterations. The resulting *λ*-stack was spectrally unmixed using the MATLAB *lsqnonneg* function, which prevents the appearance of unphysical negative coefficients. The base spectra required for unmixing were directly obtained from the deconvoled raw data, by averaging the spectra in selected spatial regions were significant spectral differences were visually apparent. A color-coded composite image showing both found constituents is shown in Fig. 5(b).

When comparing the images of regular (a) and spectral (b) ISM, we find the spatial resolution of the former to be slightly better along the horizontal (dispersion) direction. The beads in (a) appear elliptic along the polarization direction, which is to be expected. On the other hand, the beads in (b) appear round, which is due to the residual spatio-spectral crosstalk.

Nevertheless, while regular ISM offers no possibility to discriminate between the two different bead species, they can be easily identified in the spectral mode: At two positions, marked with white arrows, the presence of “TetraSpeck” beads is revealed.The retrieved spectra are shown in (c). Interestingly, the PS-Speck spectrum has its maximum at about 682 nm, which stands in contradiction with the manufacturer’s data (660 nm). However, we verified our findings using a commercial spectrometer (Ocean Optics USB4000).

**Fig. 6:**
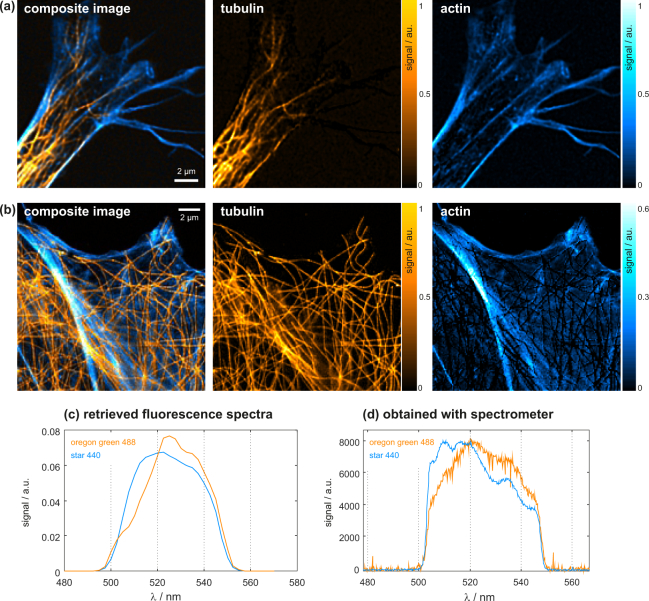
Experimental results from the imaging of double-stained HeLa cells (NA=1.4). (a), (b) show different regions within cells; 1st row: composite false-color image, tubulin (OregonGreen488) is shown in orange and actin (STAR440) in blue. 2nd, 3rd rows: individual contributions from tubulin and actin. (c) Emission spectra retrieved from the eISM measurement. (d) Emission spectra measured with a commercial spectrometer.

Results of an imaging experiment on double-stained HeLa cells are presented in Fig. 6

. The dyes used for staining the proteins actin (STAR440) and tubulin (OregonGreen488) have similar emission spectra, such that separating their contributions using color filters on the detection side would be impractical. Details about the specimen preparation are provided in the Appendix. For the experiments with biological samples we chose SLM grating periods of *P_x_* = 3 pixels in the horizontal and *P_y_* = 24 pixels in the vertical direction, resulting in a wavelength dispersion of 3.75 nm/pixel at the camera.

The figure shows false-color composite images of HeLa cell sections. The image dimensions are 15×15 μm^2^ in (a) and 16.5×16.5 μm^2^ in (b). The excitation power was on the order of 5 μW. Tubulin (stained with OregonGreen488) is shown in orange, actin (stained with STAR440) in blue. The individual spectral components are shown at the bottom of the figure. The 3D (x,y,*λ*) data stacks returned by the multi-view deconvolution algorithm are spectrally unmixed using the MATLAB *lsqnonneg* function. The required base spectra were in a first step obtained from separate eISM measurements on HeLa cells that have been exclusively stained with only one of the two dyes. In a second step, refined base spectra were directly obtained from the double-stained sample, by calculating mean values over selected cell regions, which after unmixing using the preliminary base-spectra evidently contained only one of the two components. The integrals of these refined base spectra (shown in (c)) are normalized to one. The spectral separation is successful apart from positions where the contribution from tubulin overwhelms that of actin. This is visible in the actin image of the example shown in (b). Note that the tubulin signal is about twice as strong. At these positions the reconstructed actin presence drops to zero.

Albeit less detailed, the spectra retrieved with eISM exhibit the same overall structure as those obtained with the commercial spectrometer (see Fig. 6(d)), which have been measured using single-dyed HeLa cell specimens. To obtain sufficient signal, the entire fluorescence emission of an extended region in the focal plane was coupled into the light guide of the spectrometer. The rapid signal drops below 500 nm and above 550 nm are due to the passband of the emission filter (505 nm – 545 nm).

## 5. Summary and discussion

We presented spectrally sensitive Image Scanning Microscopy, which combines the benefits of ISM, i.e. the high spatial resolution and light efficiency, with the capability of spectral sensing. The method is enabled by using a binary phase grating in the detection pupil and processing of the recorded first diffraction orders using a multi-view Lucy-Richardson algorithm. Our work can be understood as a particular implementation of hyperspectral imaging. Related algorithmic spatio-spectral unmixing techniques borrowed from tomography were quite early demonstrated for widefield imaging [32].

Despite the topical focus of this paper lies on fluorescence spectroscopy, the method should be applicable to measuring Raman spectra as well. From a functional point of view, the method presented here is related to a previous implementation of Raman-ISM [15]. The methodology, however, is notably different: While the concept introduced in [15] requires the specific hardware of a commercial Raman microscope, such as a fiber-coupled spectrometer, the method presented here is compatible with engineered ISM (eISM) as introduced in [16], i.e., it can be realized using a scanning microscope with programmable pupils in conjunction with camera detection. Using appropriate PSF designs it will be also possible to combine color-sensing as presented here with the capturing of additional information such as 3D structural data [16, 19, 25]. This particular combination has been already explored for widefield localization microscopy [30].

The phase mask we employed to enable spectral ISM is a binary Ronchi phase grating in the detection pupil. While this approach is probably one of the most intuitive and straightforward, there exist also other possibilities of introducing color sensitivity, such as using scattering discs or multimode fibers [33]. The advantage of such strategies is the possibility to achieve exquisite spectral sensitivity without introducing extreme diffraction angles or spectrometer lengths. On the other hand, very careful experimental calibration of spatially and spectrally dependent PSFs would be required, which would take the form of speckle patterns rather than the usual compact cigar-shapes.

### 5.1. The use of SLMs

Spectrally sensitive ISM can be straightforwardly realized using diffraction gratings fabricated in glass or alternative substrates. Nonetheless, we would like to briefly discuss the advantages and drawbacks of using SLMs. The liquid crystal SLM we employ in our experiments operates on one linear polarization state. Orthogonally polarized light is blocked using a polarizer. Together with the SLM’s light utilization efficiency of about 80% the total efficiency is thus merely on the order of 40%. Nevertheless, despite the high loss, using a dynamic element such as an SLM has also advantages: Most importantly, an optimal trade-off between spectral resolution and SNR can be obtained by selecting an appropriate grating period. For instance, if only well-separated spectral peaks have to be identified, a coarse grating can be displayed, thus avoiding unnecessary high dispersion. Furthermore, the ideal phase modulation depth of *π* can be set for the current central emission wavelength to optimize the diffraction efficiency. Lastly, it is also possible to split the emission light according to its polarization state and send both parts onto separate phase masks displayed on the same SLM [34–37]. Above improving the setup’s detection efficiency, this strategy offers the additional advantage of providing polarization information, which can be used to infer orientations of molecules.

The obtainable spectral resolution depends on the displayed grating period. The limiting factor is the total number of available pixels across the image of the objective’s exit pupil on the SLM. For the experiments and simulations presented in this paper we displayed gratings with a period of 3 pixels, which results in a spectral resolution of about 16 nm. By reducing the period to 2 pixels, the resolution can be further improved to about 11 nm in our current setup configuration. However, since the pupil image covers only a region of 280 pixel diameter on the SLM (the second SLM half is used for engineering the excitation pupil), the resolution could be improved to about 5 nm if entire display of our SLM were used.

### 5.2. Increasing scan speed

**Fig. 7:**
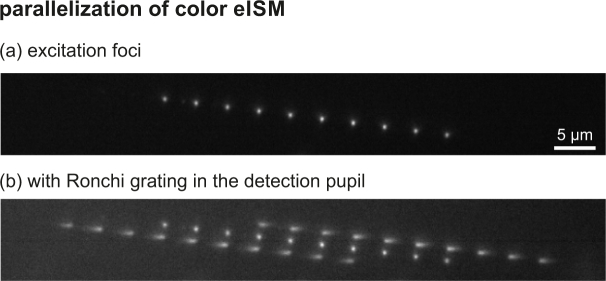
Demonstration of parallel excitation in color eISM. (a) multiple excitation foci are generated using a fan-out mask displayed on the SLM in the excitation pupil. (b) The binary phase grating in the detection pupil creates diffraction orders which are read out simultaneously. The spots showing no dispersion are the zero orders of the detection phase mask. The zero order of the excitation mask is outside the displayed area.

**Fig. 8:**
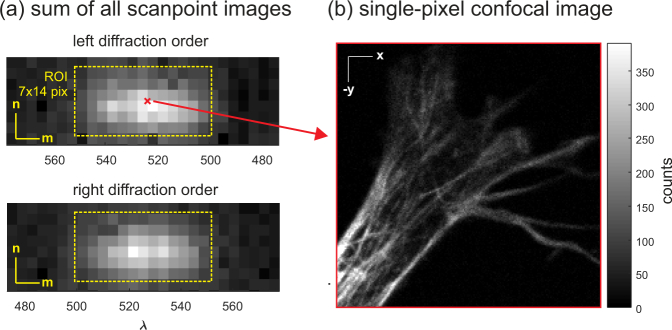
(a) The raw data consists of camera ROIs containing the diffraction orders recorded at every scanpoint. (b) Confocal image of a particular pixel.

As mentioned in the main manuscript, the scan speed provided by our current setup is quite modest. However, significantly reducing recording times is possible, even with our current camera model, since only a few lines of the sensor have to be read out. Our camera supports frame rates of more than 25 kHz for an 8-line-readout, which would enable scanning 100×100 pixel images in less than half a second. However, accessing this performance demands a different triggering scheme than currently used (camera must be master device). Another possibility of increasing the scan speed involves parallelization using a diffractive fan-out phase mask in the excitation pupil or a multi-lens array, such that many excitation spots are produced. The diffraction orders of light originating from the individual excitation sites can be interlaced as shown in Fig. 7

. The figure shows an experimental image, where 10 excitation spots have been produced by a fan-out mask displayed on the excitation side SLM. The spots are arranged along a tilted line with a spot interspacing of 4 μm. The sample is a glass slide with a thin homogeneous fluorescing layer on top. The generated fluorescence is imaged using the Ronchi phase grating in the detection path. About 50 camera rows have to be read out for the shown example, which is possible at a frame rate of about 3.2 kHz for our camera model.

## Figures and Tables

**Fig. 9:**
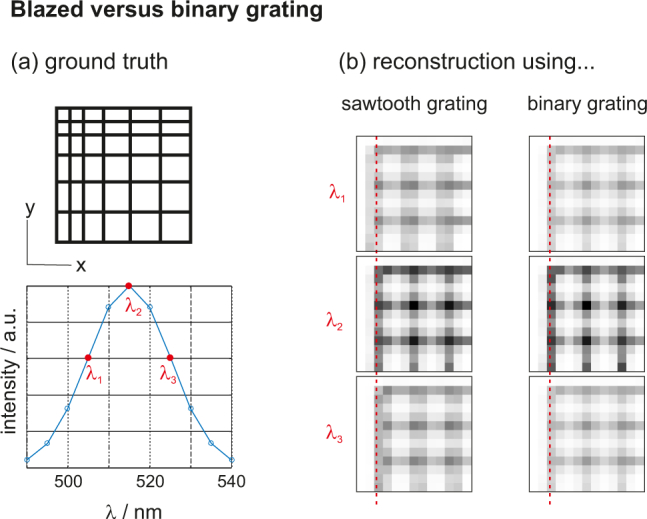
Comparing deconvolution results of data produced using blazed and binary phase gratings. The assumed object is a fluorescent 2D structure consisting of horizontal and vertical lines measuring 2.4×2.4 μm^2^. Its spatial structure and emission wavelength spectrum are shown in (a). The object estimates at three different wavelengths (*λ*_1_, *λ*_2_, *λ*_3_) after 400 deconvolution iterations are shown in (b). The estimate based on blazed grating data exhibits a wavelength-dependent positional drift, which is indicated by the red dashed line.

## Appendix

### Preparation of HeLa cells

HeLa cells were routinely cultivated in DMEM, 10% FCS, 100 IU PenStrep, 2 mM L-Glutamine and seeded onto 18x18 mm high-precision glass coverslips the day prior to fixation and staining. Cell culture reagents were from LifeTechnologies. Fixation and permeabilization were essentially performed as described in [38]. For fixation cells medium was removed and directly replaced with prefixation solution: 0.3% Glutaraldehyde , 0.25% Triton-X-100 in 1x cytoskeleton buffer (1xCB: 10 mM MES, 150 mM NaCl, 5 mM EGTA, 5 mM MgCl2, 5 mM Glucose, pH=6.1) for 2 min at 37°C. All reagents were from Sigma. Thereafter fixation was done in 2% Glutaraldehyde in 1x CB for 10 min at 37°C. Fixed cells were washed 3 x with PBS and incubated in freshly prepared 0.1% NaBH4 for 7 min. After 3 washes with PBS, cells were blocked for 15 min in 10% normal goat serum in PBS (10% NGS). Anti-a-tubulin antibody (Sigma, clone DMA1) was diluted 1:100 in 10% NGS and incubated with the cells for a 2.5 h at RT. Cells were washed 2x with PBS and incubated with sec. anti-mouse STAR 440SX (Abberior, 1:50 in 10% NGS) and Phalloidin-Oregon Green 488 (Thermo-Fisher, 1:200) for 2h at RT. Thereafter, cells were washed 3 times with PBS and embedded in ProLong Diamond mounting medium (Thermo-Fisher).

### Data processing

The raw data consists of a stack of “scanpoint images”, i.e. small images of the focal region at every scanpoint. In the case of color-sensitive eISM, each scanpoint image consists of ROIs containing the first diffraction orders of the binary grating in the detection path. The positions of the respective ROIs on the camera sensor are stored as well, such that – together with the known dispersion factor – it is possible to construct wavelength axes.

The sum of all scanpoint images, which represent the raw data of the HeLa experiment of Fig. 6, is shown in Fig. 8(a)
. A pixel with coordinates (m,n) of this sum contains the energy of an entire confocal image $I_{m,n}\left(x,y\right)$. The confocal image affiliated to the “strongest” detector pixel (marked with a red “x”) is shown in (b). The pixels lying within the yellow-framed rectangular regions are used for data reconstruction. Each rectangle measures 7×14 pixels, such that the entire raw data consist of about 5.45 Mpix.

The MV deconvolution algorithm described at the beginning of this paper requires a stack of “views” $I_{m,n}$ and their corresponding PSFs. See Ref. [19] for details about the algorithm.

Since it is difficult to measure color-dependent PSFs as needed for the deconvolution, we relied on calculated ones. This approach is feasible because phase aberrations in the microscope are measured and corrected using indirect modal wavefront sensing [39]. We simulate the PSFs for all relevant detector pixels according to Eq. (2), also taking into account vectorial effects and considering the polarizer in the detection path. The PSFs have three dimensions: x,y, and *λ*, but could be extended to three spatial dimensions as well in case x,y,z-image stacks have to be recorded. The deconvolution approach will be analogous. The wavelength axis covers the range from 500 to 550 nm in 3 nm steps, which is sufficiently small to support the spectral resolution (≈ 12 nm).

We consider two methods of processing the raw data: The first, slower approach takes the confocal images of every relevant detector pixel as individual inputs. Since the number of views can be quite high (196 in this example), this approach is comparably slow. The alternative, faster approach (which we employed to process our experimental data) merges the confocal images of pixels lying in the same column using pixel-reassignment, thus significantly reducing the number of views (to merely 28 in our example). Each merged view is calculated as:

$${\tilde{I}}_{m}\left(x,y\right)=\underset{n}{\sum }I_{m,n}\left(x,y+n\cdot a_{m}\right),$$ (3)

where *a_m_* are the column-dependent reassignment values:

$$a_{m}=\frac{1}{1+\lambda_{em}\left(m\right)/\lambda_{0}}\cdot r.$$ (4)

Note that the reassignment values depend on the column, using the respective Stokes-shifts [40]. The factor *r* denotes the ratio of effective pixel size (= real camera pixel size divided by magnification) to sampling distance. For our example the effective pixel size is 106 nm and the sampling distance 80 nm, thus r=1.3. The deconvolution algorithm performed 200 iterations and took about 10 minutes on an Intel Xeon E5-1620 CPU @3.60 GHz.

### Sawtooth versus binary grating

Figure 9
 illustrates the performance difference between using blazed and binary gratings. The ground truth of the assumed object is shown in (a) and results of the simulated imaging process in (b). Note that the color scales are inverted, i.e. black equals maximum intensity. While the spatial position of the reconstructed object depends on the wavelengths when imaging with a blazed grating (a left-to-right drift is notable when going from shorter to larger wavelengths), the features remain correctly in place when a binary grating is used.
